# 2-Methyl-1,2-benzisothia­zol-3(2*H*)-one 1,1-dioxide

**DOI:** 10.1107/S1600536808004637

**Published:** 2008-03-20

**Authors:** Waseeq Ahmad Siddiqui, Saeed Ahmad, Hamid Latif Siddiqui, Masood Parvez

**Affiliations:** a Department of Chemistry, University of Sargodha, Sargodha, Pakistan; b Department of Chemistry, University of Science and Technology, Bannu, Pakistan; c Institute of Chemistry, University of the Punjab, Lahore, Pakistan; d Department of Chemistry, The University of Calgary, 2500 University Drive NW, Calgary, Alberta, Canada T2N 1N4

## Abstract

All atoms of the title mol­ecule, C_8_H_7_NO_3_S, except the two oxide O atoms and two H atoms of the methyl group, lie on a crystallographic mirror plane. The crystal structure is stabilized by weak inter- and intra­molecular C—H⋯O hydrogen bonds.

## Related literature

For related literature, see: Hu *et al.* (2004 ▶); Kap-Sun & Nicholas (1998 ▶); Liang *et al.* (2006 ▶); Masashi *et al.* (1999 ▶); Nagasawa *et al.* (1995 ▶); Siddiqui *et al.* (2006 ▶, 2007*a* ▶,*b* ▶,*c* ▶); Siddiqui, Ahmad, Khan & Siddiqui (2007 ▶); Siddiqui, Ahmad, Khan, Siddiqui & Ahmad (2007 ▶); Siddiqui, Ahmad, Khan, Siddiqui & Parvez (2007 ▶).
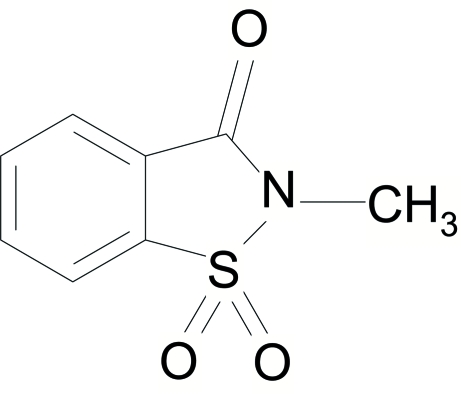


## Experimental

### 

#### Crystal data

C_8_H_7_NO_3_S
*M* *_r_* = 197.21
Monoclinic, 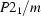

*a* = 7.463 (7) Å
*b* = 6.761 (6) Å
*c* = 8.748 (8) Å
β = 103.78 (3)°
*V* = 428.7 (7) Å^3^
*Z* = 2
Mo *K*α radiation
μ = 0.35 mm^−1^
*T* = 173 (2) K
0.12 × 0.08 × 0.07 mm

#### Data collection

Nonius KappaCCD diffractometer
Absorption correction: multi-scan (*SORTAV*; Blessing, 1997 ▶) *T*_min_ = 0.960, *T*_max_ = 0.976
1724 measured reflections
1045 independent reflections
889 reflections with *I* > 2σ(*I*)
*R*_int_ = 0.023

#### Refinement

*R*[*F* ^2^ > 2σ(*F* ^2^)] = 0.041
*wR*(*F* ^2^) = 0.106
*S* = 1.03
1045 reflections
76 parameters
H-atom parameters constrained
Δρ_max_ = 0.41 e Å^−3^
Δρ_min_ = −0.42 e Å^−3^

### 

Data collection: *COLLECT* (Hooft, 1998 ▶); cell refinement: *DENZO* (Otwinowski & Minor, 1997 ▶); data reduction: *SCALEPACK* (Otwinowski & Minor, 1997 ▶); program(s) used to solve structure: *SAPI91* (Fan, 1991 ▶); program(s) used to refine structure: *SHELXL97* (Sheldrick, 2008 ▶); molecular graphics: *ORTEPII* (Johnson, 1976 ▶); software used to prepare material for publication: *SHELXL97*.

## Supplementary Material

Crystal structure: contains datablocks global, I. DOI: 10.1107/S1600536808004637/lh2597sup1.cif

Structure factors: contains datablocks I. DOI: 10.1107/S1600536808004637/lh2597Isup2.hkl

Additional supplementary materials:  crystallographic information; 3D view; checkCIF report

## Figures and Tables

**Table 1:** Hydrogen-bond geometry (Å, °)

*D*—H⋯*A*	*D*—H	H⋯*A*	*D*⋯*A*	*D*—H⋯*A*
C8—H8*A*⋯O1	0.96	2.49	2.869 (4)	104
C2—H2⋯O1^i^	0.95	2.29	3.227 (4)	169
C8—H8*B*⋯O2^ii^	0.96	2.49	3.358 (3)	151

Symmetry codes: (i) 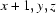; (ii) 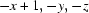.

**Fig. 1.**
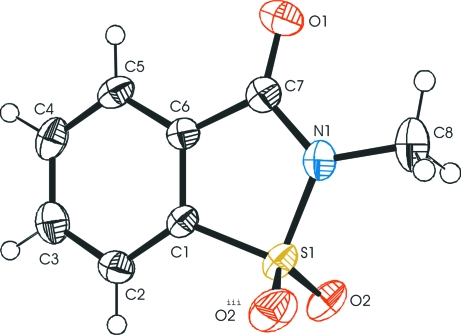
ORTEPII (Johnson, 1976) drawing of (I) with displacement ellipsoids plotted at 50% probability level. Symmetry code: (iii) x, -y + 1/2, z.

## supplementary crystallographic information

### Comment

Benzisothiazolone-1,1-dioxide is part of a class of heterocycles which has been investigated in pharmaceutical research (Kap-Sun & Nicholas, 1998). 1,2-benzisothiazole-3-one 1,1-dioxide (saccharin) has been widely incorporated into a variety of biologically active compounds. It has been identified as an important molecular component in various classes of 5-HTla antagonists, analgesics and human mast cell tryptase inhibitors (Liang *et al.*, 2006). In particular, N-substituted derivatives, e.g with *N*-hydroxy and *N*-alkyl substituents, have shown important biological activites (Nagasawa *et al.*, 1995). Among *N*-alkyl derivatives, various synthetic routes have been reported for the synthesis of the title compound involving ionic liquids and free radical mechanisms (Hu *et al.*, 2004; Masashi *et al.*, 1999). In continuation of our research on the synthesis of 1,2-benzothiazine 1,1-dioxide derivatives, we have in addtion, embarked on the synthesis of benzisothiazole derivatives (Siddiqui *et al.*, 2006; Siddiqui *et al.*, 2007*a*,b,c; Siddiqui, Ahmad, Khan & Siddiqui, 2007; Siddiqui, Ahmad, Khan, Siddiqui & Ahmad, 2007; Siddiqui, Ahmad, Khan, Siddiqui & Parvez, 2007). Herein, we report the synthesis and crystal structure of the title compound, (I).

With the exception atoms O2 and H8B, all atoms of the molecule of (I) (Fig. 1) lie on a crystallographic mirror plane. The benzisothiazole moiety is exactly planar. The molecular dimensions are in accord with the corresponding dimensions reported in similar structures (Siddiqui *et al.*, 2007*a*-c; Siddiqui, Ahmad, Khan, Siddiqui & Parvez, 2007). The structure is stabilized by one intramolecular and two intermolecular interactions of the type C—H···O (details are in Table).

### Experimental

Saccharin (2.0 g, 11.0 mmol.) was added to a solution of sodium hydroxide (0.875 g, 22.0 mmol.) in distilled water (25 ml) under constant stirring to give a transparent solution. A solution of dimethylsulfate (2.08 ml, 22.0 mmol.) in methanol (10.0 ml) was then added dropwise over 2 minutes. Precipitates started appearing within 5 minutes and stirring was continued for 20 min. at room temperature. The precipitates were filtered, washed with cold water and dried (343 K) to get 1.75 g of (I) (8.9 mmol. 81%). Recrystallization Solvent: CHCl_3_. The solution was subjected to slow evaporation at 313 K to obtain colourless crystals.

### Refinement

H-atoms bonded were included in the refinements at geometrically idealized positions with aromatic and methyl C—H distances 0.95 and 0.96 Å, respectively, and *U*_iso_ = 1.2 times *U*_eq_ of the atoms to which they were bonded. The final difference map was free of any chemically significant features.

### Crystal data

**Table:**

C_8_H_7_NO_3_S	*F*_000_ = 204
*M**_r_* = 197.21	*D*_x_ = 1.528 Mg m^−^^3^
Monoclinic, *P*2_1_/*m*	Mo *K*α radiation λ = 0.71073 Å
Hall symbol: -P 2yb	Cell parameters from 1724 reflections
*a* = 7.463 (7) Å	θ = 3.2–27.4º
*b* = 6.761 (6) Å	µ = 0.35 mm^−^^1^
*c* = 8.748 (8) Å	*T* = 173 (2) K
β = 103.78 (3)º	Prism, colorless
*V* = 428.7 (7) Å^3^	0.12 × 0.08 × 0.07 mm
*Z* = 2	

### Data collection

**Table:**

Nonius KappaCCD diffractometer	1045 independent reflections
Radiation source: fine-focus sealed tube	889 reflections with *I* > 2σ(*I*)
Monochromator: graphite	*R*_int_ = 0.023
*T* = 173(2) K	θ_max_ = 27.4º
ω and φ scans	θ_min_ = 3.2º
Absorption correction: multi-scan(SORTAV; Blessing, 1997)	*h* = −9→9
*T*_min_ = 0.960, *T*_max_ = 0.976	*k* = −8→8
1724 measured reflections	*l* = −11→11

### Refinement

**Table:**

Refinement on *F*^2^	Secondary atom site location: difference Fourier map
Least-squares matrix: full	Hydrogen site location: inferred from neighbouring sites
*R*[*F*^2^ > 2σ(*F*^2^)] = 0.041	H-atom parameters constrained
*wR*(*F*^2^) = 0.106	*w* = 1/[σ^2^(*F*_o_^2^) + (0.0455*P*)^2^ + 0.2966*P*] where *P* = (*F*_o_^2^ + 2*F*_c_^2^)/3
*S* = 1.03	(Δ/σ)_max_ < 0.001
1045 reflections	Δρ_max_ = 0.41 e Å^−^^3^
76 parameters	Δρ_min_ = −0.42 e Å^−^^3^
Primary atom site location: structure-invariant direct methods	Extinction correction: none

### Special details

**Table:**

Geometry. All e.s.d.'s (except the e.s.d. in the dihedral angle between two l.s. planes) are estimated using the full covariance matrix. The cell e.s.d.'s are taken into account individually in the estimation of e.s.d.'s in distances, angles and torsion angles; correlations between e.s.d.'s in cell parameters are only used when they are defined by crystal symmetry. An approximate (isotropic) treatment of cell e.s.d.'s is used for estimating e.s.d.'s involving l.s. planes.

### Fractional atomic coordinates and isotropic or equivalent isotropic displacement parameters (Å^2^)

**Table:**

	*x*	*y*	*z*	*U*_iso_*/*U*_eq_	
S1	0.68037 (10)	0.2500	0.26611 (7)	0.0314 (2)	
O1	0.2425 (3)	0.2500	0.4038 (3)	0.0416 (5)	
O2	0.7324 (2)	0.0698 (2)	0.20266 (16)	0.0445 (4)	
N1	0.4534 (3)	0.2500	0.2520 (3)	0.0314 (5)	
C1	0.7314 (4)	0.2500	0.4722 (3)	0.0254 (5)	
C2	0.9043 (4)	0.2500	0.5750 (3)	0.0337 (6)	
H2	1.0141	0.2500	0.5381	0.040*	
C3	0.9090 (4)	0.2500	0.7337 (3)	0.0403 (7)	
H3	1.0249	0.2500	0.8079	0.048*	
C4	0.7486 (4)	0.2500	0.7873 (3)	0.0379 (7)	
H4	0.7566	0.2500	0.8974	0.045*	
C5	0.5767 (4)	0.2500	0.6832 (3)	0.0308 (6)	
H5	0.4670	0.2500	0.7203	0.037*	
C6	0.5690 (3)	0.2500	0.5235 (3)	0.0251 (5)	
C7	0.4012 (4)	0.2500	0.3931 (3)	0.0289 (6)	
C8	0.3218 (5)	0.2500	0.0986 (3)	0.0455 (8)	
H8A	0.1982	0.2500	0.1130	0.055*	
H8B	0.3405	0.1341	0.0410	0.055*	

### Atomic displacement parameters (Å^2^)

**Table:**

	*U*^11^	*U*^22^	*U*^33^	*U*^12^	*U*^13^	*U*^23^
S1	0.0415 (4)	0.0319 (4)	0.0237 (3)	0.000	0.0133 (3)	0.000
O1	0.0284 (10)	0.0449 (13)	0.0521 (13)	0.000	0.0107 (9)	0.000
O2	0.0587 (10)	0.0435 (9)	0.0369 (8)	0.0058 (7)	0.0223 (7)	−0.0101 (7)
N1	0.0355 (12)	0.0301 (12)	0.0266 (11)	0.000	0.0031 (9)	0.000
C1	0.0319 (13)	0.0222 (12)	0.0237 (12)	0.000	0.0099 (10)	0.000
C2	0.0276 (13)	0.0367 (16)	0.0371 (14)	0.000	0.0085 (11)	0.000
C3	0.0402 (16)	0.0417 (17)	0.0340 (15)	0.000	−0.0010 (12)	0.000
C4	0.0553 (18)	0.0343 (16)	0.0238 (13)	0.000	0.0087 (12)	0.000
C5	0.0392 (15)	0.0257 (13)	0.0324 (14)	0.000	0.0181 (12)	0.000
C6	0.0280 (12)	0.0189 (12)	0.0297 (13)	0.000	0.0093 (10)	0.000
C7	0.0330 (14)	0.0221 (13)	0.0320 (13)	0.000	0.0086 (11)	0.000
C8	0.0551 (19)	0.0452 (19)	0.0284 (15)	0.000	−0.0053 (13)	0.000

### Geometric parameters (Å, °)

**Table:**

S1—O2^i^	1.430 (2)	C2—H2	0.9500
S1—O2	1.430 (2)	C3—C4	1.386 (4)
S1—N1	1.668 (3)	C3—H3	0.9500
S1—C1	1.752 (3)	C4—C5	1.385 (4)
O1—C7	1.211 (3)	C4—H4	0.9500
N1—C7	1.380 (4)	C5—C6	1.384 (4)
N1—C8	1.462 (4)	C5—H5	0.9500
C1—C2	1.386 (4)	C6—C7	1.479 (4)
C1—C6	1.389 (4)	C8—H8A	0.9600
C2—C3	1.380 (4)	C8—H8B	0.9600
			
O2^i^—S1—O2	116.79 (14)	C4—C3—H3	119.2
O2^i^—S1—N1	109.63 (8)	C5—C4—C3	121.1 (3)
O2—S1—N1	109.63 (8)	C5—C4—H4	119.5
O2^i^—S1—C1	112.76 (8)	C3—C4—H4	119.5
O2—S1—C1	112.76 (8)	C6—C5—C4	118.2 (2)
N1—S1—C1	92.54 (12)	C6—C5—H5	120.9
C7—N1—C8	123.3 (2)	C4—C5—H5	120.9
C7—N1—S1	115.6 (2)	C5—C6—C1	119.7 (2)
C8—N1—S1	121.1 (2)	C5—C6—C7	127.0 (2)
C2—C1—C6	122.7 (2)	C1—C6—C7	113.2 (2)
C2—C1—S1	127.5 (2)	O1—C7—N1	124.0 (3)
C6—C1—S1	109.9 (2)	O1—C7—C6	127.2 (3)
C3—C2—C1	116.7 (3)	N1—C7—C6	108.8 (2)
C3—C2—H2	121.6	N1—C8—H8A	109.6
C1—C2—H2	121.6	N1—C8—H8B	109.4
C2—C3—C4	121.6 (3)	H8A—C8—H8B	109.5
C2—C3—H3	119.2		
			
O2^i^—S1—N1—C7	−115.28 (8)	C3—C4—C5—C6	0.000 (1)
O2—S1—N1—C7	115.28 (8)	C4—C5—C6—C1	0.0
C1—S1—N1—C7	0.0	C4—C5—C6—C7	180.0
O2^i^—S1—N1—C8	64.72 (8)	C2—C1—C6—C5	0.0
O2—S1—N1—C8	−64.72 (8)	S1—C1—C6—C5	180.0
C1—S1—N1—C8	180.0	C2—C1—C6—C7	180.0
O2^i^—S1—C1—C2	−67.46 (9)	S1—C1—C6—C7	0.0
O2—S1—C1—C2	67.46 (9)	C8—N1—C7—O1	0.0
N1—S1—C1—C2	180.0	S1—N1—C7—O1	180.0
O2^i^—S1—C1—C6	112.54 (9)	C8—N1—C7—C6	180.0
O2—S1—C1—C6	−112.54 (9)	S1—N1—C7—C6	0.0
N1—S1—C1—C6	0.0	C5—C6—C7—O1	0.0
C6—C1—C2—C3	0.0	C1—C6—C7—O1	180.0
S1—C1—C2—C3	180.0	C5—C6—C7—N1	180.0
C1—C2—C3—C4	0.000 (1)	C1—C6—C7—N1	0.0
C2—C3—C4—C5	0.000 (1)		

Symmetry codes: (i) *x*, −*y*+1/2, *z*.

### Hydrogen-bond geometry (Å, °)

**Table:**

*D*—H···*A*	*D*—H	H···*A*	*D*···*A*	*D*—H···*A*
C8—H8A···O1	0.96	2.49	2.869 (4)	104
C2—H2···O1^ii^	0.95	2.29	3.227 (4)	169
C8—H8B···O2^iii^	0.96	2.49	3.358 (3)	151

Symmetry codes: (ii) *x*+1, *y*, *z*; (iii) −*x*+1, −*y*, −*z*.
